# Folate Deficiency Increased Microglial Amyloid-β Phagocytosis via the RAGE Receptor in Chronic Unpredictable Mild-Stress Rat and BV2 Cells

**DOI:** 10.3390/nu15163501

**Published:** 2023-08-08

**Authors:** Junting Fan, Zewei Ma, Yunqin Zheng, Meilin Zhang, Li Huang, Huan Liu

**Affiliations:** 1 Department of Nutrition and Food Science, School of Public Health, Tianjin Medical University, Tianjin 300070, China; 2 Tianjin Key Laboratory of Environment, Nutrition, and Public Health, Center for International Collaborative Research on Environment, Nutrition and Public Health, Tianjin 300070, China

**Keywords:** folate deficiency, microglia, β-amyloid protein (Aβ), phagocytosis, chronic unpredictable mild stress (CUMS), receptor for advanced glycation end products RAGE

## Abstract

Depression is often considered one of the prevalent neuropsychiatric symptoms of Alzheimer’s disease (AD). β-amyloid (Aβ) metabolism disorders and impaired microglia phagocytosis are potential pathological mechanisms between depression and AD. Folate deficiency (FD) is a risk factor for depression and AD. In this study, we used a chronic unpredictable mild stress (CUMS) rat model and a model of Aβ phagocytosis by BV2 cells to explore the potential mechanisms by which FD affects depression and AD. The results revealed that FD exacerbated depressive behavior and activated microglia in CUMS rats, leading to an increase in intracellular Aβ and phagocytosis-related receptors for advanced glycation end products (RAGE). Then, in vitro results showed that the expression of the RAGE receptor and M2 phenotype marker (CD206) were upregulated by FD treatment in BV2 cells, leading to an increase in Aβ phagocytosis. However, there was no significant difference in the expression of toll-like receptor 4 (TLR4) and clathrin heavy chain (CHC). Furthermore, when using the RAGE-specific inhibitor FPS-ZM1, there was no significant difference in Aβ uptake between folate-normal (FN) and FD BV2 cell groups. In conclusion, these findings suggest FD may promote microglia phagocytosis Aβ via regulating the expression of RAGE or microglia phenotype under Aβ treatment.

## 1. Introduction

Chronic stress can induce neuropsychiatric disorders, including depression [1], and disrupt cognitive processes and neuroplasticity [2]. Depression is a leading cause of disability, which imposes a major financial burden on society [3]. Importantly, depression is frequently detected in the early stages of Alzheimer’s disease (AD) and is associated with the progression of the disease [4]. Epidemiological evidence suggests that individuals with depression experience a decline in cognitive function [5], and a three-year prospective study reported that 57% of depressed, non-demented individuals developed dementia [6]. Depression manifestation prior to the onset of AD is closely linked with the development of AD, even when the first symptoms occur 25 years before AD onset. These symptoms may serve as early indicators of the dementing process [5,7]. Therefore, there was a strong link between depression and AD.

Activated microglia induced by amyloid clumped close to β-amyloid (Aβ) in the protracted preclinical stage of AD diagnosis [8]. Microglia recognized Aβ as a foreign substance and removed Aβ by phagocytosis in nondementia individuals [8], which suggested that pathologic events in the preclinical phase of AD were Aβ deposition and microglial activation. Serum Aβ concentration had a positive association with depression severity in depression patients [9]. Furthermore, in our previous study, chronic unpredictable mild stress (CUMS) elevatored Aβ deposition and activated microglia [10]. Microglia play crucial roles in synaptic plasticity and neurogenesis, which activation cause AD and major depressive disorder [11]. Furthermore, the study found that the density of microglial activation was positively correlated with the severity of depressive episodes [12]. When microglia were activated, the expression of surface receptors for binding Aβ, such as the toll-like receptor 4 (TLR4), the receptor for advanced glycosylation end products (RAGE), and complement receptors also upregulated [13]. Downregulation of RAGE and TLR4, among others, explains the impaired Aβ phagocytosis in microglia [14,15]. Moreover, in our previous study, CUMS induce upregulation of RAGE and TLR4 to promote Aβ deposition in microglia [10]. Thus, it is crucial to explore the connection between the expression of RAGE and TLR4 and microglia phagocytosis function in depression.

Deviations in microglial homeostasis induced by long periods of microglial activation due to AD could promote depression [16]. Activated microglia can manifest as two polarized types with divergent phenotypes and roles, the classically activated M1 type and the alternatively activated M2 type, depending on the microenvironment [17]. A study conducted on animals suggested that M2-type microglia with the ability to phagocytose Aβ in 6 months old mice transform into M1-type phenotypes at 18 months old, which is linked to Aβ oligomer accumulation [18]. Therefore, further research is necessary to investigate the timing and process of microglial transition into different phenotypes.

A previous study reported that folate deficiency (FD) elevated hippocampal amyloid precursor protein (APP) and Aβ protein levels, and these increases were ameliorated by folate supplementation through increasing DNA methyltransferase activity [19]. Folate affects neurotransmitter synthesis and neurons’ structural elements, so its deficiency results in cognitive dysfunction and depression [20,21,22,23]. Several studies associated lower folate levels or higher Hcy levels with a higher risk of AD or depression [24,25,26]. In vivo, folate showed antidepression-like effects and anti-inflammatory effects in CUMS rats [27]. FD enhanced microglia immune responses in rats with brain injury [28]. Interestingly, supplementing with oral 500 μg/d folate improved the antidepressant effects of fluoxetine in depressed women [29], which suggested that folate supplementation could ameliorate depression. Given that, folate deficiency may act through increased Aβ levels and activate microglia to worsen depression.

Therefore, the objective of this study was to further explore the impact of FD on depressive-like behavior, as well as investigate the possible mechanisms involved. In order to explore the possible mechanisms, the effect of FD on exacerbating depressive-like behavior by amyloid deposition and microglia activation was examined using both the CUMS rat model, which is the most popular and widely considered as one of the most accurate models of depression [30,31,32], and conducted in vitro experiments on BV2 cells. The impact of FD on the uptake of Aβ via RAGE or TLR4 receptors and the regulation of microglia M1/M2 phenotypes were also investigated.

## 2. Methods

### 2.1. Animals

Male Sprague–Dawley (SD) rats, which were 3 months old (180 ± 20 g), were obtained from SPF (Beijing) Biotechnology Co., Ltd. (China) and maintained in a standard environment at 21 ± 1 °C temperature, 55 ± 5% humidity and 12 h light/12 h dark cycle, as well as free access to food and water. After a week of habituating to the standard-control environment, SD rats were randomly assigned into three groups (n = 10/group): the control group (Control), CUMS model group (CUMS), CUMS combined with folate deficiency group (CUMS+FD). The CUMS+FD group was fed a folate-deficient diet (0.0 mg/kg), while all other groups were fed a normal folate diet (2.1 mg/kg). The animal experimental study, with the approval of the Animal Ethics and Welfare Committee of Tianjin Nankai Hospital (NKYY-DWLL-2020-180), was conducted.

### 2.2. Chronic Unpredictable Mild Stress

The program for CUMS is based on Li H et al. [33] and as previously described [34]. Rats were randomly subjected to a different mild stressor each day to minimize the possibility of predicting occurrence of stimulation by the rats. After 12 weeks of continuous stimulation, the CUMS model was successfully established, as evidenced by significant differences in behavioral experiments between the CUMS group, CUMS+FD group, and the control group. The control group did not undergo any CUMS stimulation throughout the entire process.

At the end of 12 weeks of intervention, three group rats were euthanized via CO_2_ inhalation after an overnight fast. Blood samples and tissues were collected, serum was obtained by centrifugation, and brain tissues were fixed in 4% (*w*/*v*) paraformaldehyde for histopathological staining or snap frozen in liquid nitrogen and stored at −80 °C for further biochemical assays.

### 2.3. Behavioral Tests

#### 2.3.1. Open-Field Test (OFT)

To assess exploratory and locomotor activity, the rats were placed in the center of an open field arena (length, 62.5 cm × width, 74 cm × height, 51 cm). They were given 5 min to freely explore the arena; during this time, the movement and time were monitored via a video tracking system (ANY-maze software 5.3). Between the trials, the box was thoroughly cleaned with 75% ethanol in an attempt to remove any residual olfactory cues. The environment was kept quiet during the experiment.

#### 2.3.2. Elevated-Plus Maze Test (EPM)

The EPM has been widely used to measure anxiety-like behaviors in rodents. Each rat was placed in the center area (10 × 10 cm, 40 cm high walls), facing a closed arm consisting of two opposing open arms/closed arms (length, 50 × width, 10 cm), and allowed to explore the maze freely for a period of 5 min while being video recorded with the ANY maze system.

### 2.4. Enzyme-Linked Immunosorbent Assay (ELISA)

The concentration of Aβ1-40 and Aβ1-42 in rat brain tissues of each group were detected with a commercial Aβ1-40 and Aβ1-42 ELISA kits (Jiangsu Meimian Industrial Co., Ltd., Yancheng, China). Prior to the assay, rat brain tissues were accurately weighed and processed, in summary, by adding PBS at the ratio of 1:9 by volume of weight, homogenizing, and then centrifuging at 5000× *g* for 5–10 min to prepare the supernatant. The standard curve was then prepared according to the manual procedure, following the standards provided. Absorbance was measured at 450 nm with a microplate reader. Finally, the protein concentration of the brain tissue supernatant was determined by using a bicinchoninic acid (BCA) protein assay kit (Sparkjade, Qingdao, China), and the Aβ1-40 and Aβ1-42 concentrations in the brain tissue were quantified according to the manufacturer’s instructions.

### 2.5. Chemiluminescent Immunoassay (CLIA)

Folate levels in rat brain tissue were measured using a competitive protein binding assay. The measurements were performed with an automated chemiluminescence system (Siemens Immune 2000XPI, Berlin, Germany), following the manufacturer’s instructions. The system exhibited a detection sensitivity limit of 0.8 ng/mL for all types of folates. The concentration of Hcy in brain tissue was assayed using Auto-Chemistry Analyzer (CS-T300, DIRUI, Changchun, China) with a sensitivity value of a limit of detection of 0.33 μmol/L, and finally, protein quantification was performed.

### 2.6. Cell Culture and Treatment

BV2 microglial cells (derived from Raf/Myc-immortalized murine neonatal microglial) were purchased from Procell, China. In brief, the cells were cultured in DMEM supplemented with 10% FBS and 1% penicillin/streptomycin, incubated at 37 °C with 5% CO_2_ and 95% air in a humidified atmosphere [35].

Preparation of oligomeric Aβ: Aβ25-35 powder (AnaSpec, Fremont, CA, USA) was dissolved in DMSO to prepare a 1.0 mmol/L stock solution, frozen at −20 °C, then diluted with cell culture medium to a final concentration of 5 μmol/L and incubating at 4 °C for 24 h. Before use, Aβ25-35 were centrifuged at 14,000× *g* for 10 min to remove fibrous and highly aggregated forms.

BV2 cells were divided into folate-normal group (FN, 10 μmol/L), folate-deficient group (FD, 0 μmol/L), folate-normal + Aβ group (FN + Aβ), folate-deficient + Aβ group (FD + Aβ), and Aβ25-35 concentrations in Aβ group were 5 μmol/L. The final intervention time was determined according to the modeling time. Then, RAGE receptor was inhibited with FPS-ZM1 to observe the effect of folate deficiency on Aβ25-35 uptake by BV2 cells. BV2 cells were divided into four groups during this stage: FN + Aβ25-35 group, FD + Aβ25-35 group, FN + FPS-ZM1 + Aβ25-35 group, and FD + FPS-ZM1 + Aβ25-35 group. The concentration of FPS-ZM1 in the inhibitor group was 100 nmol/L.

### 2.7. Cell Viability Assay

The viability of cells was determined using a 3-(4,5-dimethylthiazol-2-yl)-5-(3-carboxymethoxyphenyl)-2-(4-sulfophenyl)-2H-tetrazolium (MTS) colorimetric assay. Briefly, 10^4^ cells were seeded into 96-well plates and incubated overnight for cell attachment. After hatching, all cell culture media were replaced with DMEM basal medium, in which the final concentration of Aβ25-35 was 5 μmol/L in the uptake Aβ group and cultured for 6, 12, 36, 24, 36, and 48 h, respectively, and then 1 mg/mL MTS reagent was added to each well and incubated for 4 h at 37 °C in a 5% CO_2_ environment. The absorbance of the solution at 490 nm was measured with a microplate reader (BioTek Instruments, Winooski, VT, USA), and the survival rate (%) of the control cells was calculated.

### 2.8. Western Blotting

Western blotting is used to detect protein expression. The cells were homogenized in RIPA buffer and then incubated on ice for 30 min. After centrifugation at 13,000 rpm for 15 min at 4 °C, the BCA protein quantification was carried out. Equal amounts of protein were separated by electrophoresis on 10% sodium dodecyl sulfate–polyacrylamide gels and transferred to polyvinylidene difluoride membranes (PVDF; Millipore, Billerica, MA, USA), and the membranes were blocked with 5% skim milk for 1 h at 25 °C. Subsequently, the membranes were placed with mouse anti-RAGE, TLR4, and CHC (1:1000; Abcam, Cambridge, England) primary antibodies overnight at 4 °C. The membranes were then incubated with secondary antibodies (horseradish peroxidase (HRP)-conjugated anti-rabbit IgG; HRP-conjugated anti-mouse IgG; 1:2000; CST, Danvers, MA, USA) for 1 h at room temperature. Proteins were then detected with chemiluminescent reagents and visualized with the ChemiDocTM XRS + Imaging System (Bio-Rad, Hercules, CA, USA). Densitometry was performed using Image j 1.4.3.67 to quantify protein levels.

### 2.9. Immunofluorescence

Activation of microglia in rat brains was detected by immunofluorescent labeling. The paraffin sections were first dewaxed in xylene and then successively immersed in 100%, 90%, 75%, and 50% ethanol and distilled water. The sections were disposed of in 3% H_2_O_2_ for 10 min at 25 °C, repaired with sodium citrate-EDTA antigen solution (Beyotime, Shanghai, China), and then blocked with goat serum for 1 h at 37 °C. The sections were incubated with primary antibody (rabbit anti-Iba-1, 1:100, Abcam) overnight at 4 °C. Thereafter, sections were washed with PBST and then incubated with TRITC (fluorescein)-conjugated goat anti-rabbit secondary antibody (1:100; Zhongshan Jinqiao Biotechnology, Beijing, China) for 1 h at 25 °C in the dark. Cell nuclei were stained with DAPI before mounting.

Aβ precipitation and expression of RAGE and TLR4 in microglia were determined by immunofluorescence double-labeling in a similar way, but the primary antibody was changed to a mixture of Iba-1 and Aβ1-40/Aβ1-42 or a mixture of Iba-1 and RAGE/TLR4 (1:100, Abcam), and the secondary antibody was changed to a mixture of murine and rabbit, which are FITC-conjugated goat anti-mouse secondary antibody (1:100, Sparkjade) and TRITC-conjugated anti-rabbit secondary antibody (1:100, Zhongshan Jinqiao Biotechnology, Beijing, China).

The uptake of Aβ25-35 by BV2 cells in vitro was determined using immunofluorescence. Slides coated with poly-L-lysine (0.1 mg/mL) were placed in 24-well plates. BV2 cells were cultured and adjusted to 5 × 10^5^ cells/mL, fluorescently labeled Aβ25-35 (Fluor 488-labeled, Purchased from AnaSpec, USA) was added, fixed in 4% paraformaldehyde for 30 min at 25 °C in the dark, and then incubated with 10% goat serum blocking solution for 1 h. The cell slides were removed, diluted cell membrane staining solution was added dropwise, and incubated for 15 min at 37 °C. Finally, DAPI containing an anti-fluorescence quenching blocking solution was added, and the expression intensity was observed under a fluorescence microscope. For immunofluorescence detection of BV2 cell activation typing and RAGE expression, the steps were similar, requiring replacement of the cell membrane staining solution with iNOS and CD206 primary antibody, which serve as markers for M1 and M2 polarization of microglia, and RAGE primary antibody, incubation overnight, followed by the corresponding incubation of secondary antibodies. The positive cells were observed using a fluorescent microscope (IX81; Olympus, Tokyo, Japan) and analyzed with Image Pro Plus 6.0 software.

### 2.10. Statistical Analysis

Statistical analysis was performed using SPSS 25.0 and ImageJ 2.1.0. Results are expressed as mean ± SD. Differences were evaluated by one-way analysis of variance (ANOVA) followed by a Least Significant Difference (LSD) multiple range test. A significance level of *p* < 0.05 was used to determine statistical significance.

## 3. Results

### 3.1. Folate Deficiency Exacerbated CUMS-Induced Depression-Like Behavior

**Figure 1:**
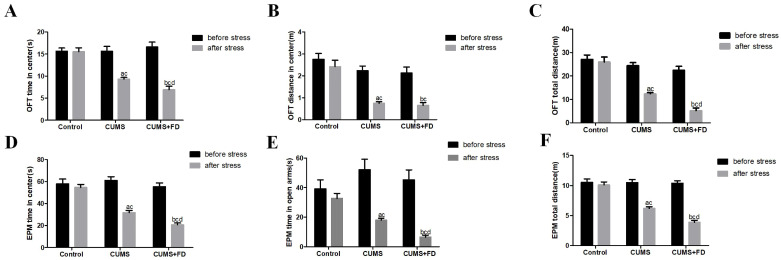
Effects of CUMS and folate deficiency on depressive behavior in rats. (**A**) OFT time in center (s). (**B**) OFT distance in center (m). (**C**) OFT total distance(m). (**D**) EPM time in center (s). (**E**) EPM time in open arm (s). (**F**) EPM total distance (m). All data are presented as the mean ± SD. ^a^ *p* < 0.05, compared with the CUMS group before stress before the intervention; ^b^ *p* < 0.05, compared with the CUMS+FD group before the stress intervention; ^c^ *p* < 0.05, compared with the control group after the stress intervention; ^d^ *p* < 0.05, compared with the CUMS group after the stress intervention (n = 10).

We employed EPM and the OFT to evaluate depression-like behavior. As shown in Figure 1, following a 12-week chronic stress intervention, the distance in a central area, the time spent in a central area, and the total distance of the OFT in CUMS and CUMS+FD groups were significantly decreased compared to before the intervention (*p* < 0.05) and lower than in the control group (*p* < 0.05).Compared to CUMS group, the CUMS+FD group showed a significant decrease in the time spent on the open arms in the EPM, as well as the time spent in the central area and the total distance traveled in EPM (*p* < 0.05).

### 3.2. Folate Deficiency Reduced Brain Folate and Increased Brain Homocysteine in CUMS Rats

**Figure 2:**
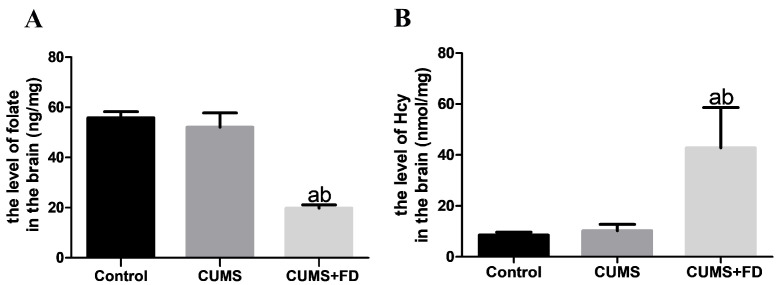
Folate and homocysteine contents of brain tissues of rats in each group. The levels of Folate (**A**) and homocysteine (**B**) in brain. ^a^ *p* < 0.05, compared with control group; ^b^ *p* < 0.05, compared with CUMS group (n = 5).

After 12 weeks of CUMS modeling and FD intervention, brain tissue folate concentration decreased in the CUMS+FD group, and there was a statistical difference between CUMS and control groups (*p* < 0.05). The CUMS+FD group had higher brain homocysteine levels than the CUMS and control groups, a difference that was statistically significant (*p* < 0.05) (Figure 2).

### 3.3. Folate Deficiency Increased Aβ Levels in the Brain of CUMS Rats

**Figure 3:**
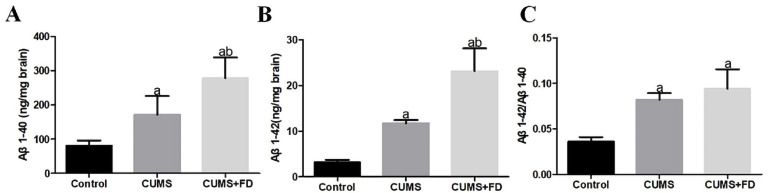
Effect of folate deficiency on brain tissue Aβ1-42 and Aβ1-40 in CUMS rats. (**A**) Brain tissue Aβ1-40 levels. (**B**) Brain tissue Aβ1-42 levels. (**C**) Brain tissue Aβ1-42/Aβ1-40. ^a^ *p* < 0.05, compared with control group; ^b^ *p* < 0.05, compared with CUMS group (n = 5).

According to Figure 3, compared to the control group, the levels of Aβ1-40 and Aβ1-42 in the brain of rats in the CUMS group were found to be higher, and the ratio of Aβ1-42 to Aβ1-40 in the CUMS group and the CUMS+FD group also increased markedly (*p* < 0.05). The levels of Aβ1-40 and Aβ1-42 in the brain tissue of rats in the CUMS+FD group were further increased compared to the other two groups, and the difference was statistically significant (*p* < 0.05). However, the ratio of Aβ1-42 to Aβ1-40 in the CUMS+FD group had no significant difference with the CUMS group.

### 3.4. Folate Deficiency Enhanced Microglia Activation and Aβ Deposition on Microglia in CUMS Rats

**Figure 4:**
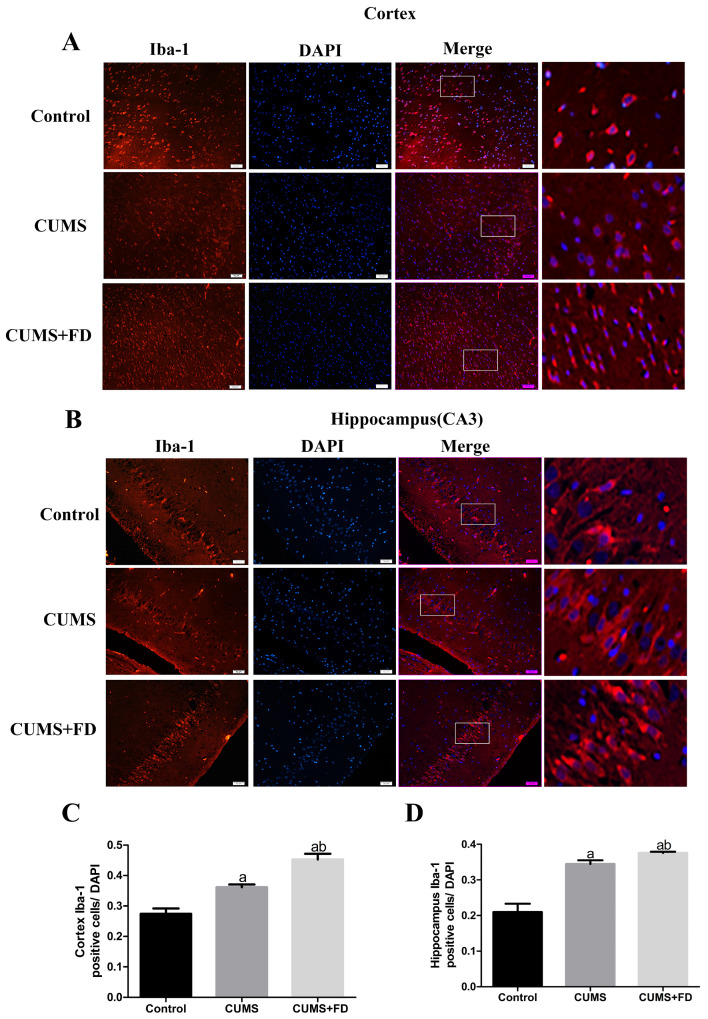
Effect of folate deficiency on microglia activation in CUMS rats. (**A**) Single-label Iba-1 plot of cortex (Iba-1 in red, DAPI in blue). (**B**) Single-labeled Iba-1 map of hippocampal CA3 region (Iba-1 in red, DAPI in blue). (**C**) The number of Iba-1 positive cells/DAPI cells in the cortex. (**D**) The number of Iba-1 positive cells/DAPI cells in the hippocampal CA3 region. ^a^ *p* < 0.05, compared with the control group; ^b^ *p* < 0.05, compared with the CUMS group (n = 5, scale bar = 50 μm).

To investigate the potential response of cortex and hippocampal (CA3) microglia to CUMS treatment alone or in combination with FD, we conducted immunostaining for Iba-1, a specific marker for microglia, which allowed us to assess the pathological changes of microglia in the ipsilateral hemisphere. As shown in Figure 4, compared to the control group, the CUMS group had substantially more Iba-1 positive cells in the cortex (Figure 4A,C) and CA3 (Figure 4B,D) following a 12-week intervention (*p* < 0.05). Additionally, compared to the control and CUMS groups, the combination of CUMS and FD intervention significantly enhanced the number of Iba-1 positive cells in the cortex and CA3 (*p* < 0.05).

**Figure 5:**
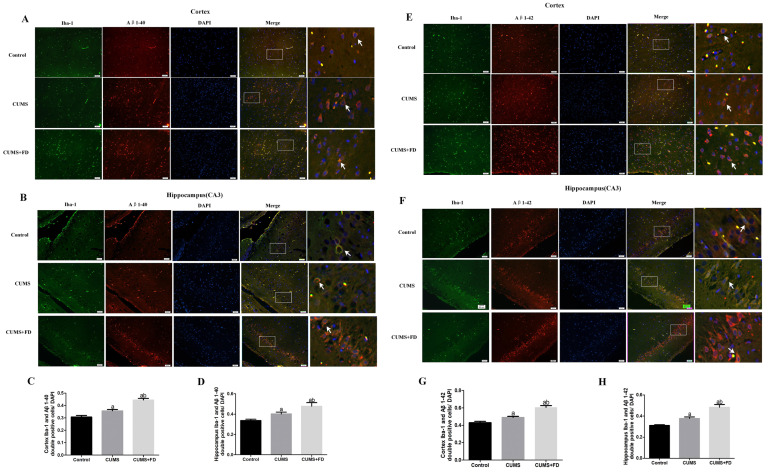
Effect of folate deficiency on Aβ levels in microglia of CUMS rats. (**A**,**B**) are Co-localization fluorescence plots of Iba-1 with Aβ1-40 in cortex and hippocampal CA3 regions. Merge image shown the positive. Each right-hand column depicts a magnified image of the rectangular region of the corresponding image in the left column, and the arrows marked Iba-1 co-localized with Aβ1-40 cells. (**C**,**D**) Resultant plots of Iba-1 with Aβ1-40 positive cell count/DAPI cell count in cortex and hippocampal CA3 regions. (**E**,**F**) Co-localization fluorescence plots of Iba-1 with Aβ1-42 in cortex and hippocampal CA3 regions, and the arrows marked Iba-1 co-localized with Aβ1-42 cells. (**G**,**H**) Resultant plots of Iba-1 with Aβ1-42 positive cell count/DAPI cell count in cortex and hippocampal CA3 regions. ^a^ *p* < 0.05, compared with control group; ^b^ *p* < 0.05, compared with CUMS group (n = 5, scale bar = 50 μm).

Aβ1-42 and Aβ1-40 antibodies identifying for Aβ deposits and Iba-1 immunostainings for microglia were carried out to see if the deposition of Aβ1-42 and Aβ1-40 was related to microglia activation in cortex and CA3 following 12 weeks of FD intervention. Aβ1-40 and Iba-1 co-localized positive cells were found in significantly higher numbers in the cortex and CA3 of the CUMS group compared to the control group (*p* < 0.05) (Figure 5A–D), whereas they were found in significantly higher numbers in the cortex and CA3 of the CUMS+FD group compared to the CUMS group (*p* < 0.05). The expression results of Aβ1-42 showed the same trend as the data in Figure 5E–H.

### 3.5. Effect of Folate Deficiency and Aβ25-35 on the Viability of BV2 Cells

**Figure 6:**
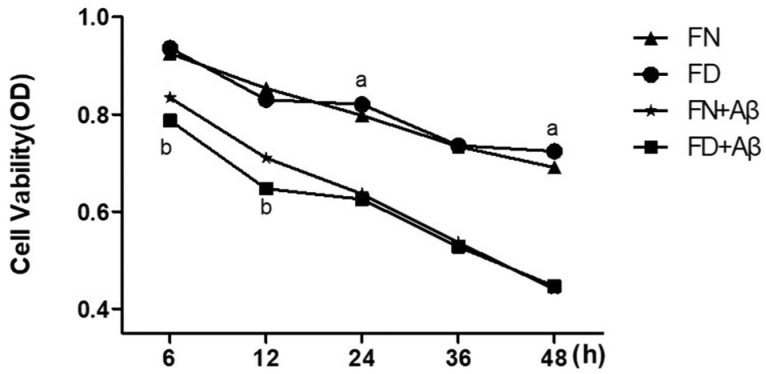
Cellular activity at different times of Aβ25-35 intervention. ^a^ *p* < 0.05, compared to FN group; ^b^ *p* < 0.05, compared to FN+Aβ group.

According to our in vivo experiments, the depressive state caused by CUMS is connected with the pathological feature of AD, Aβ. APP is hydrolyzed to Aβ1-40 and Aβ1-42 by γ-secretase [36], of which Aβ1-42 is the main component in the formation of senile plaques and causes neuronal death and cognitive decline [37]. Aβ25-35 is the active peptide fragment of Aβ1-42, and its oligomeric form is the main active site causing neurotoxicity [38]. In the early stages of AD, there were microglia inflammation and high levels of soluble Aβ1-42 [39]. Furthermore, soluble Aβ oligomers are responsible for depressive-like behavior [40,41]. These studies suggest that depression, as an early stage of AD [42], may be caused by pathological changes in soluble Aβ. Thus, we chose Aβ25-35 oligomeric, the active peptide fragment of oligomeric Aβ1-42, for in vitro experiments. To ensure that BV2 cells perform phagocytic function, oligomeric Aβ25-35 at 5 μmol/L, which is less microglia-cytotoxic and does not affect microglia immune function, is chosen for in vitro experiments. The cell viability will be influenced by the nutritional status of culture media [43]; thus, we only analyzed the differences in cell viability between groups rather than the changing trends over time. We found that the cell activity of BV2 cells decreased when folic acid levels were normal or deficient with Aβ25-35 intervention. This decrease was statistically significant compared to both the FN and FD groups (*p* < 0.05). At 6 and 12 h, the FD+Aβ group exhibited lower activity compared to the FN+Aβ group, and this difference was statistically significant (*p* < 0.05) (Figure 6). At 24 and 48 h, the FD group had higher cell activity, and this difference was statistically significant compared to the FN group (*p* < 0.05). At 36 h, the BV2 cell activity did not differ significantly between the FD and FN groups, as well as between the FN+Aβ and FD+Aβ groups (*p* > 0.05). In conclusion, to avoid the possible effect of cell viability on phagocytosis in BV2 cells, subsequent in vitro studies used 36 h as the Aβ25-35 intervention time.

### 3.6. Effect of Folate Deficiency on Activation and Polarization of BV2 Cells

**Figure 7:**
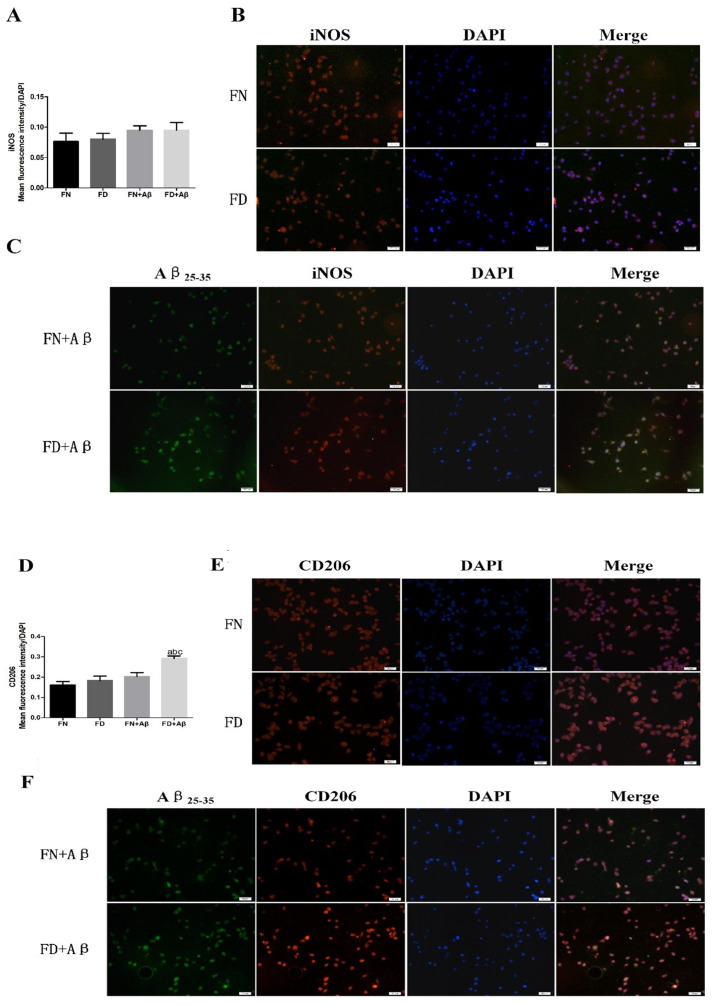
Effect of folate deficiency on BV2 cell typing. (**A**) is a plot of the results of the average fluorescence value of iNOS/number of DAPI cells. (**B**,**C**) are iNOS fluorescence plots, co-localization fluorescence plots of exogenous fluorescent marker Aβ25-35 with iNOS (Aβ25-35 in green, iNOS in red, DAPI in blue). (**D**) is a plot of the average fluorescence value of CD206/number of DAPI cells that results in plots. (**E**,**F**) are CD206 fluorescence plots, and exogenous fluorescence labeled Aβ25-35 co-localized with CD206 fluorescence plots (Aβ25-35 in green, CD206 in red, and DAPI in blue). ^a^ *p* < 0.05, compared with FN group; ^b^ *p* < 0.05, compared with FD group; ^c^ *p* < 0.05, compared with FN+Aβ group (n = 3, scale bar = 50 μm).

The results showed that FD significantly enhances microglia stimulation and exacerbates Aβ deposition on microglia. To further investigate the impact of FD and Aβ on microglia activation and polarization, we conducted assays at the BV2 cell level. We used immunofluorescence staining to measure the expression of fluorescently labeled Aβ25-35-stimulated microglia phenotypic markers in BV2 cells at 36 h. FD combined with Aβ25-35 did not significantly increase the fluorescence intensity of iNOS (M1 marker) (Figure 7A,C) but significantly increased CD206 (M2 marker) compared with the other three groups (*p* < 0.05) (Figure 7D,F). In addition, there was no difference between the FN and FD groups in the absence of fluorescence-labeled Aβ25-35 intervention (Figure 7B,E).

### 3.7. Effect of Folate Deficiency on Aβ-Related Receptors on Microglia

**Figure 8:**
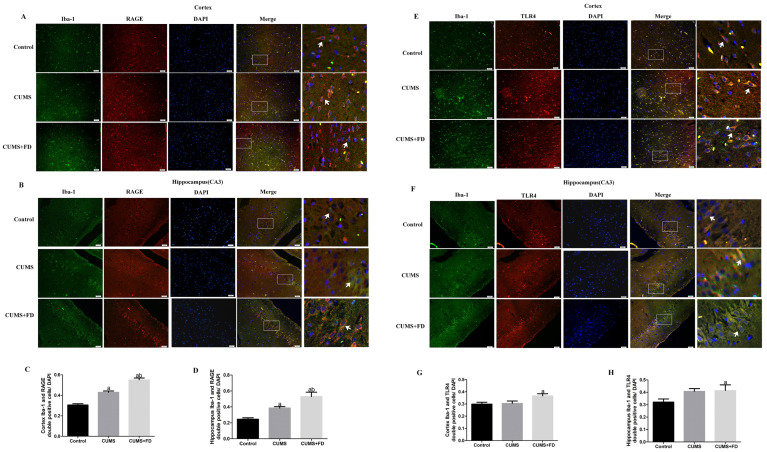
Effect of folate deficiency on RAGE, TLR4 receptors on microglia membranes in CUMS rats. Cortex and hippocampal CA3 regions Iba-1 co-localized with RAGE fluorescence plots are the upper and lower parts of (**A**,**B**). Merge image shown the positive. Each right-hand column depicts a magnified image of the rectangular region of the corresponding image in the left column, and the arrows marked Iba-1 co-localized with RAGE cells. (**C**,**D**) are the cortex and hippocampal CA3 regions Iba-1 co-localized with RAGE positive cell count/DAPI cell count result statistics plots. Cortex and hippocampal CA3 regions Iba-1 co-localized with TLR4 fluorescence plots are the two parts of (**E**,**F**), the arrows marked Iba-1 co-localized with TLR4 cells. (**G**,**H**) are the cortex and hippocampal CA3 regions Iba-1 with TLR4 positive cell count/DAPI cell count histogram. ^a^ *p* < 0.05, compared with control group; ^b^ *p* < 0.05, compared with CUMS group (n = 5, scale bar = 50 μm).

Double immunofluorescence labeling for RAGE and Iba-1, as well as TLR4 and Iba-1, was carried out to see if the effect of folate deficit on microglia absorption of Aβ was receptor-related. As shown in Figure 8, the number of RAGE and Iba-1 double-positive cells in the cerebral cortex and CA3 was significantly increased in the CUMS group compared with the control group (*p* < 0.05), and this increase was further seen in the CUMS+FD group compared to the CUMS group (*p* < 0.05). Although there was no discernible difference in the number of TLR4 and Iba-1 double-positive cells in the CUMS group and the control group, there was a considerable increase in these cells in the CUMS+FD group (*p* < 0.05).

**Figure 9:**
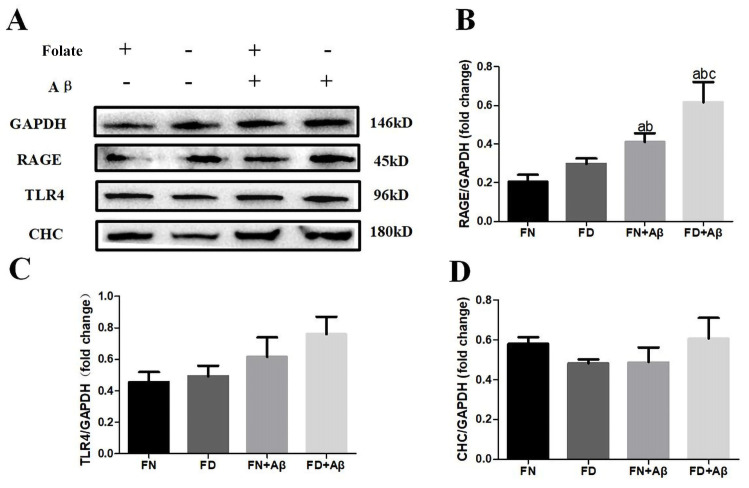
Effect of folate deficiency on RAGE, TLR4 receptor, and lattice protein expression on BV2 cell membranes. (**A**) Western blot was used to detect the expression of RAGE, TLR4, and CHC protein for each group. (**B**–**D**) represent the results of Immunoblot analysis conducted to measure the protein levels of RAGE, TLR4, and CHC in the brain. ^a^ *p* < 0.05, compared with FN group; ^b^ *p* < 0.05, compared with FD group; ^c^ *p* < 0.05, compared with FN+Aβ group (n = 3).

**Figure 10:**
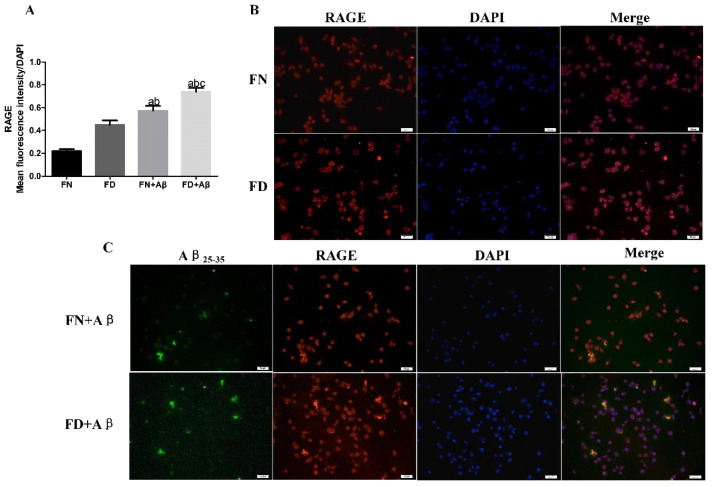
Effect of folate deficiency on RAGE receptor expression on BV2 cell membrane. (**A**) Results of the mean fluorescence intensity of RAGE/number of DAPI cells statistics. (**B**) RAGE fluorescence plot of BV2 cells (RAGE is red, DAPI is blue). (**C**) Co-localized fluorescence plot of exogenous fluorescent markers Aβ25-35 and RAGE (Aβ25-35 is green, RAGE in red, DAPI in blue). ^a^ *p* < 0.05, compared with FN group; ^b^ *p* < 0.05, compared with FD group; ^c^ *p* < 0.05, compared with FN+Aβ group (n = 3, scale bar = 50 μm).

Based on the observation from in vivo experiments that intracellular levels of Aβ correlate with RAGE receptors on microglia, we proceeded to further validate the effect of FD on Aβ-related receptors and the endocytic pathway in in vitro experiments. Immunofluorescence staining and Western blot assayed protein expression of Aβ-related receptor markers and endocytic pathway at 36 h. Under FD or Aβ25-35 intervention, the expression of CHC, a significant component protein contributing to endocytosis, did not show any statistically significant difference in BV2 cells. Similarly, there was no noticeable variation in TLR4 expression (Figure 9A,C,D). However, RAGE expression was significantly higher in the FD+ Aβ group compared to the other three groups (*p* < 0.05). Additionally, RAGE expression was increased in the FN+Aβ group when compared to the FN or FD groups individually (*p* < 0.05) (Figure 9A,B). These findings were further supported by cellular immunofluorescence assays, where the FD+ Aβ group exhibited significantly higher RAGE fluorescence intensity in BV2 cells than the other three groups (*p* < 0.05) (Figure 10).

### 3.8. Effect of Folate Deficiency on RAGE Expression and Uptake of Aβ25-35 in FPS-ZM1-Intervened BV2 Cells

**Figure 11:**
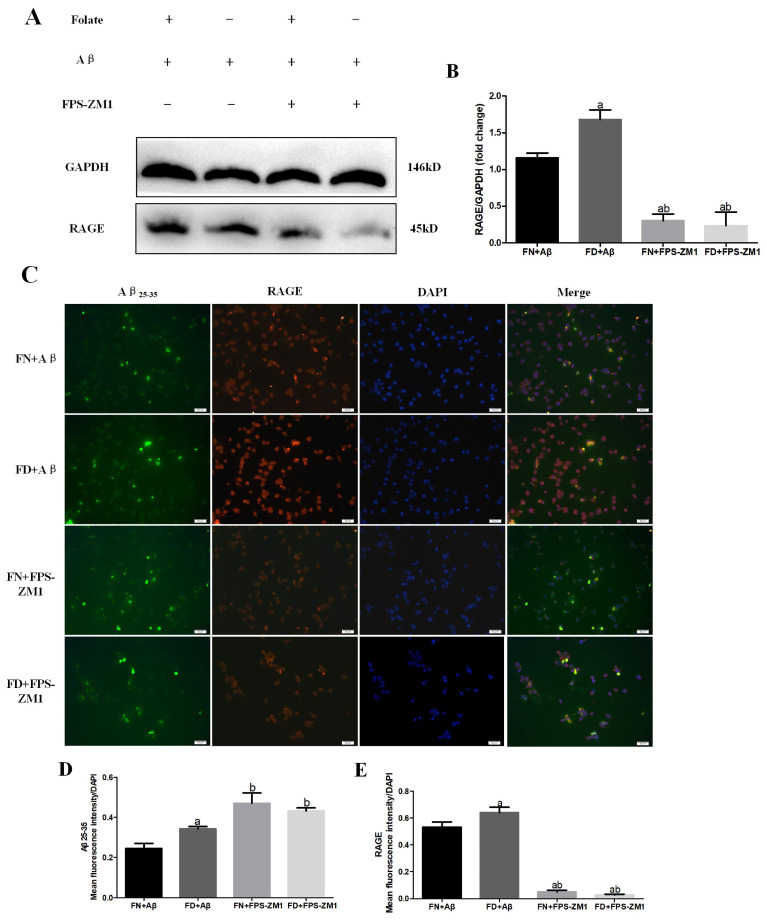
Effect of folate deficiency on RAGE expression and phagocytosis of Aβ in FPS-ZM1-intervened BV2 cells. (**A**) RAGE immunoblot images. (**B**) Results of immunoblot analysis of protein levels in RAGE. (**C**) Co-localized fluorescence plot of exogenously fluorescently labeled Aβ25-35 and RAGE (Aβ25-35 in green, RAGE in red, DAPI in blue). (**D**,**E**) are Aβ25-35, RAGE mean fluorescence value/DAPI cell number histogram. ^a^ *p* < 0.05, compared with FN+Aβ group; ^b^ *p* < 0.05, compared with FD+Aβ group (n = 3, scale bar = 50 μm).

The effect of FPS-ZM1, a specific RAGE inhibitor, on intracellular Aβ25-35 fluorescence intensity in BV2 under folate deficit was investigated in order to further explore the potential function of the RAGE pathway in FD-induced changes in Aβ25-35 uptake by BV2 cells. The immunofluorescence analysis (Figure 11C–E) showed that the fluorescence intensity of RAGE-positive cells raised by Aβ25-35 combined with or without folate deficiency was significantly decreased by FPS-ZM1 administration compared with FN+Aβ and FD+Aβ groups (*p* < 0.05). However, the fluorescence intensity of intracellular Aβ25-35 was markedly elevated after FPS-ZM1 administration compared with FN+Aβ and FD+Aβ groups (*p* < 0.05). FPS-ZM1 blocked the expression of RAGE in BV2 after Aβ25-35 combined with or without FD treatment, compared with FN+Aβ and FD+Aβ groups (*p* < 0.05) (Figure 11A,B).

## 4. Discussion

Depression, as an early stage of AD, could influence the development of AD [42,44], but the potential pathological mechanisms between depression and AD are still unclear [45]. Disturbances in Aβ metabolism and alterations in microglia phagocytosis are central events in the development of AD, and interestingly similar elevated levels of brain inflammation and Aβ also found in depressed individuals [46]. Thus, it is crucial to explore the common risk factors of AD and depression for alterations in Aβ and microglia phagocytosis in the preclinical phase of AD. As one of the common risk factors for depression and AD, FD exacerbated depressive behavior and elevated Aβ deposition in microglia in CUMS rats in this study. Moreover, it was validated that FD could promote microglia phagocytosis of Aβ via the RAGE receptor.

Depression, as an early stage of AD, has pathological changes associated with AD, such as alterations in Aβ and microglia phagocytosis [44,47]. After 12 weeks of intervention, FD exacerbated the depressive behavior, which was caused by CUMS. Several other studies have also revealed the effects of FD on depression, consistent with our study [48,49]. As a reliable predictor of amyloid load in the brain and biomarkers in AD [50,51], the ratio of Aβ1-42 to Aβ1-40 was elevated in CUMS rats, which means CUMS caused AD-related pathological changes in depressive-behavior rats. The same alterations were found in our previous study [10]. In this study, Aβ deposition induced by CUMS was further elevated by FD. It has been suggested that one of the reasons for the increased Aβ content in brain tissue by FD may relate to the upregulation of Aβ production pathway BACE1 [52]. In the preclinical phase of AD, microglia recognize Aβ as a foreign substance and remove it through phagocytosis after being activated. Our study is consistent with previous findings that FD activates microglia [53,54]. Additionally, folate supplementation has been shown to inhibit microglial activation [55] and reduce the inflammatory response caused by microglia or CUMS [27,56]. Our in vivo study revealed that FD not only exacerbates Aβ deposition on microglia but also activates microglia in the cortex and hippocampal regions of CUMS rats. The significant content of exogenous Aβ in BV2 cells cultured in FD demonstrated that FD may activate microglia to phagocytose Aβ.

Different degrees of microglia activation are associated with distinct functions, as indicated by the M1/M2 phenotype of microglia. With aging and increasing levels of Aβ, the activation of microglia in AD mice undergoes a dynamic process [39]. In the brains of 6-month-old AD mice, microglia are mainly M2, but as age and Aβ accumulation progress, microglia transition towards the M1 phenotype and exhibit reduced phagocytosis [57]. This suggests that in the early stages of AD, microglia display the M2 phenotype and contribute to microglial phagocytosis [57]. In our study, we observed that FD may enhance microglia-mediated phagocytosis of Aβ in the brains of CUMS rats in vivo, and this effect may be associated with the phenotype. After incubation with oligomeric Aβ25-35 for 36 h, expression of the M2 phenotype marker CD206 and intracellular Aβ levels were elevated in folate-deficient BV2 cells, indicating that FD in combination with Aβ regulates microglia differentiation toward the M2 phenotype rather than M1. In summary, folate deficiency enhances microglia phagocytose Aβ, which is likely associated with the M2 phenotype.

Microglia also phagocytose Aβ through Aβ-related mechanisms such as RAGE, scavenger receptors, and TLR. These receptors have been shown to bind to ligands such as soluble Aβ, which presumably contribute to neurodegeneration [58]. It has also been shown that RAGE helps epithelial cells to take up the oligomer Aβ1-42 [59]. Meanwhile, upregulation of RAGE may be associated with increased Hcy levels brought about by FD [60] or indirectly increase Aβ levels. There are high levels of RAGE expression around age spots [61], and our results found that FD increased RAGE expression, thus upregulating Aβ levels in microglia both in vivo and in vitro. After RAGE was inhibited, no difference in Aβ25-35 content was found between folate normal and folate deficiency groups of BV2 cells, further validating that FD promotes microglia phagocytosis of Aβ, possibly by regulating RAGE expression.

However, elevated Aβ25-35 levels have been observed in BV2 cells after RAGE inhibition at various folate levels. When Aβ25-35 cannot be phagocytosed by RAGE, it can accumulate in the culture medium and is highly susceptible to forming fibrillar Aβ at 37 °C. Fibrillar Aβ can potentially be phagocytosed via TLR4 or other receptors [62]. We presume that the elevation of Aβ phagocytosis after RAGE inhibition may be a result of structural changes in Aβ and alterations in other receptors. Furthermore, a decrease in microglia inflammatory response promotes microglia phagocytosis of Aβ [63,64,65,66]. RAGE is involved in the activation of multiple inflammatory pathways within microglia [67], and FPS-ZM1 could attenuate RAGE-induced neuroinflammation in microglia [68]. Therefore, promoting cytophagy of Aβ after FPS-ZM1 intervention also may be caused by attenuating the inflammatory response of microglia due to RAGE.

TLRs were considered to play a key role in the signaling of Aβ and trigger a cascade reaction that leads to phagocytosis and clearance of Aβ [69]. Microglia require a specific structure of Aβ aggregates to phagocytose them through TLR4 receptors. Fibrillar Aβ directly interacts with TLR4 and facilitates phagocytosis in microglia [70]. CD14, a member of the TLRs family, shows a 20-fold higher affinity for fibrillar Aβ1-42 compared to non-fibrillar Aβ1-42 [71]. Additionally, BV2 cells only exhibit phagocytosis of oligomeric Aβ through TLR4 after LPS (lipopolysaccharide) stimulation [69]. In our study, we did not observe any effect of FD (presumably referring to a specific treatment or condition) on TLR4 expression in BV2 cells, possibly due to the in vitro nature of the Aβ structure analysis.

It has been observed that oligomeric Aβ1-42 can be phagocytosed by SH-SY5Y cells through clathrin-mediated endocytosis [72]. However, our study did not find any evidence that FD regulates the expression of microglia latticework proteins or affects microglia phagocytosis of Aβ. Unfortunately, we did not examine the expression of TLR4 and other receptors after RAGE inhibition. Although we explored microglia phagocytosis of soluble Aβ, this is a limitation given that there is some immunity of microglia to Aβ deposition, which we did not explore. Further studies are needed in the future to clarify the mechanism by which FD enhances Aβ phagocytosis.

In conclusion, FD aggravated the depressive behavior of CUMS rats and increased the level of microglial intracellular Aβ and microglial RAGE expression in the brain. FD may promote microglia phagocytosis Aβ via regulating the expression of RAGE or the microglia phenotype.

## Data Availability

The data presented in this study are available upon request from the corresponding author.
